# Free-electron creation at the 60° twin boundary in Bi_2_Te_3_

**DOI:** 10.1038/ncomms12449

**Published:** 2016-08-16

**Authors:** Kwang-Chon Kim, Joohwi Lee, Byung Kyu Kim, Won Young Choi, Hye Jung Chang, Sung Ok Won, Beomjin Kwon, Seong Keun Kim, Dow-Bin Hyun, Hyun Jae Kim, Hyun Cheol Koo, Jung-Hae Choi, Dong-Ik Kim, Jin-Sang Kim, Seung-Hyub Baek

**Affiliations:** 1 Center for Electronic Materials, Korea Institute of Science and Technology, Seoul 136-791, Republic of Korea; 2 School of Electrical and Electronic Engineering, Yonsei University, Seoul 120-749, Republic of Korea; 3 Department of Materials Science and Engineering, Kyoto University, Kyoto 606-8501, Japan; 4 High Temperature Energy Materials Research Center, Korea Institute of Science and Technology, Seoul 136-791, Republic of Korea; 5 KU-KIST Graduate School of Converging Science and Technology, Korea University, Seoul 136-701, Republic of Korea; 6 Center for Spintronics, Korea Institute of Science and Technology, Seoul 136-791, Republic of Korea; 7 Advanced Analysis Center, Korea Institute of Science and Technology, Seoul 136-791, Republic of Korea; 8 Department of Nanomaterials, Korea University of Science and Technology, Daejeon 305-333, Republic of Korea

## Abstract

Interfaces, such as grain boundaries in a solid material, are excellent regions to explore novel properties that emerge as the result of local symmetry-breaking. For instance, at the interface of a layered-chalcogenide material, the potential reconfiguration of the atoms at the boundaries can lead to a significant modification of the electronic properties because of their complex atomic bonding structure. Here, we report the experimental observation of an electron source at 60° twin boundaries in Bi_2_Te_3_, a representative layered-chalcogenide material. First-principles calculations reveal that the modification of the interatomic distance at the 60° twin boundary to accommodate structural misfits can alter the electronic structure of Bi_2_Te_3_. The change in the electronic structure generates occupied states within the original bandgap in a favourable condition to create carriers and enlarges the density-of-states near the conduction band minimum. The present work provides insight into the various transport behaviours of thermoelectrics and topological insulators.

## Abstract (web summary)

Grain boundaries in polycrystalline materials may offer the opportunity to explore physical phenomena that do not normally occur within the crystal grains. Here, the authors show that twin boundaries in Bi_2_Te_3_ works as an electron supply for the whole bulk material.

## Graphical abstract

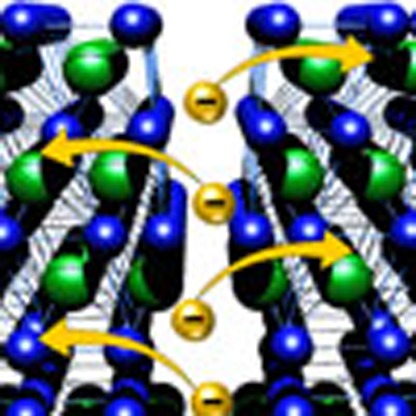

A grain boundary is the interface between two crystalline grains with different orientations in polycrystalline solids^1^,^2^. The periodic arrangement of atoms is broken at the grain boundary, and structural modifications such as strain, atomic displacement, non-stoichiometry and atomic bonding changes are usually accommodated within the grain boundary area. Given that the physical properties of a material are directly relevant to the atomic bonding structure, the grain boundary has different properties from the grain. Moreover, there are, in principle, unlimited ways to form grain boundaries with five degrees of freedom, and each of them can have its own unique physical property because of the particular atomic structure^1^.

In this regard, grain boundaries can provide a promising platform to explore emerging phenomena that do not exist within the grain^3^. Recently, a substantial modification of physical properties was demonstrated in twin boundaries—a particular type of grain boundary with a mirror symmetry—of complex oxides^1^,^2^. For example, the insulating, multiferroic BiFeO_3_ shows electrical conductivity at 71°, 109° and 180° twin boundaries^4^,^5^, abnormal photovoltaic effect^6^ and large magnetoresistance at the 109° twin boundary^7^. This is attributed to the large modification of the electronic structure by atomic displacement depending on the type of twin boundary: each twin wall provides its own way of atomic bonding distortion, resulting in distinctly emerging properties. The twin boundary, a coherent and low-energy interface, is relatively stable compared with a normal grain boundary. Such a stability of the twin boundary can make it easier to explore potentially emergent functionalities and promising to integrate with real devices with reliable performances.

Therefore, it is crucial to study this issue—searching for unexpected properties from a twin boundary arising from the local atomic misfits—in Bi_2_Te_3_, as a representative of layered-chalcogenide materials, which is the basic model system for both room-temperature thermoelectricity and topological insulator behaviour^8^,^9^. These phenomena are directly related to transport properties such as carrier density and mobility. Thermoelectric properties such as Seebeck coefficient, electrical conductivity and thermal conductivity are strongly interrelated as a function of the carrier density^10^,^11^. Thus, there exists a carrier density (∼10^19^ cm^−3^) to maximize the thermoelectric performance^10^. It is reported that the grain boundary can play a key role in further improvements of thermoelectric properties^12^,^13^,^14^. Grain boundaries in nano-grain Bi_2_Te_3_ alloys can significantly suppress thermal conductivity by effectively scattering the phonons over the electrical carriers. To observe the topological insulator phenomenon, it is critical to reduce the carrier density to as low a level as possible^15^,^16^,^17^,^18^,^19^; otherwise, the surface transport by a topological insulator is surpassed by the bulk conduction.

Here, we experimentally show that the 60° twin boundary in Bi_2_Te_3_ creates electrons: it works as an electron source for the bulk Bi_2_Te_3_. We observe that the bulk carrier density proportionally increases with the length of the 60° twin boundary, while the mobility decreases. The theoretical calculation reveals that the modified interatomic distance at the boundary leads to the production of an extra occupied state within the band gap.

## Results

### Epitaxial growth of (001) Bi_2_Te_3_ films

To investigate the properties of a grain or twin boundary, it is essential to create well-defined, single-type boundaries within a single-crystal sample. Bi-crystals, where two single crystals are joined at controlled angles and orientations, can provide such a platform. However, as only a single line of the grain boundary can be formed in a bi-crystal, this approach is valid only when the grain boundary acts as a strong limiting factor of the properties concerned^20^,^21^. Otherwise, microscopic analyses, for example, scanning probe microscopy, may be possible, but this is often very challenging and limited. Moreover, for a Bi_2_Te_3_ material, it is very difficult to fabricate well-defined and clean grain boundaries using a bi-crystal because it is very brittle as a layered material with van der Waals bonding along the *c*-axis. Cracks and point defects are easily developed during polishing.

**Figure 1:**
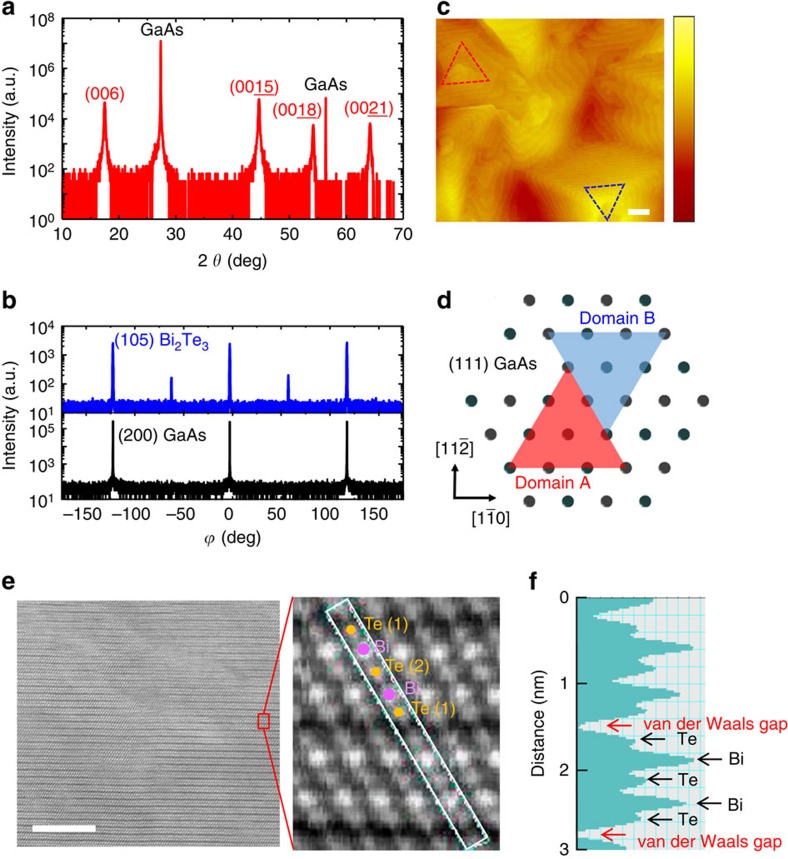
Structural characterization of Bi_2_Te_3_ films. (**a**) X-ray diffraction out-of-plane *θ*–2*θ* scan of the Bi_2_Te_3_/GaAs heterostructure. (**b**) The in-plane *ϕ* scan with (105) Bi_2_Te_3_ and (200) GaAs diffraction peaks. Two domains of Bi_2_Te_3_ are observed. (**c**) The surface morphology of a 200 nm-thick Bi_2_Te_3_ thin film measured by AFM. The flat terraces and steps of quintuple layers (QLs) are observed. The scale bar within the AFM image and the colour scale bar represent 1 μm and 10 nm, respectively. The root-mean-square surface roughness is 2.2 nm. (**d**) The schematic illustration of two Bi_2_Te_3_ domains with a 60° rotation on the GaAs (111) surface. Because of the three-fold symmetry of the [001] axis, each domain is described as a triangle with different colours (red and blue). (**e**) High-resolution cross-sectional high-angle annular dark field scanning tunnelling electron microscopy (HAADF-STEM) results. The high-magnification image clearly shows the sequence of Bi and Te atoms, which are labelled as Te (1)–Bi–Te (2)–Bi–Te (1) atoms (Te: orange, Bi: purple). The scale bar is 10 nm. (**f**) The line scan across the Bi_2_Te_3_ QLs in the white box area in (**e**) shows the different intensity of Bi and Te atoms, and the van der Waals gap.

A key aspect of the present study is our ability to engineer the domain structure of epitaxial Bi_2_Te_3_ thin films to create high-density, single-type twin boundaries. Bi_2_Te_3_ films were grown on (111) GaAs substrates using the metal-organic chemical vapour deposition technique^22^,^23^. The crystalline quality of the Bi_2_Te_3_ thin films are analysed by four-circle high-resolution X-ray diffraction. Figure 1a shows the out-of-plane *θ*–2*θ* scan of the Bi_2_Te_3_ film on (111) GaAs substrates, where *θ* is the angle between the incident beam and the crystallographic planes. The X-ray diffraction pattern shows that the Bi_2_Te_3_ film is purely (001)-oriented along the surface normal direction. The out-of-plane lattice is 30.4 Å, which is consistent with bulk Bi_2_Te_3_. Figure 1b shows the azimuthal *ϕ* scan of the (105) plane of the Bi_2_Te_3_ film as well as the (200) plane of the GaAs substrate, where *ϕ* is the azimuthal angle of the sample with respect to the sample normal. For the cubic GaAs substrate, three (200) peaks are obtained with 120° intervals because of the three-fold symmetry along the [111] direction. On the other hand, for rhombohedral Bi_2_Te_3_, six (105) peaks are observed with 60° intervals. Considering the rhombohedral symmetry of Bi_2_Te_3_ with a space group of R
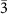
m, we can deduce that the Bi_2_Te_3_ film on (111) GaAs has an in-plane domain structure, where each domain is oriented with a 60° rotation^24^,^25^. The intensity difference of the (105) peaks shows that one domain has a larger population than the other. Additionally, chemical analyses by atomic electron spectroscopy and X-ray photoelectron spectroscopy reveal that our Bi_2_Te_3_ thin films are stoichiometric within the detection limits, as shown in Supplementary Fig. 1.

The surface morphology of the Bi_2_Te_3_ films is characterized by atomic force microscopy (AFM), as shown in Fig. 1c. The surface is atomically smooth with a clear step-and-terrace structure. The morphology indicates that the film growth is governed by the two-dimensional nucleation and growth mode. More importantly, it is conspicuous that a triangular morphology with two different orientations evolves on the surface as marked by blue dotted triangles. This indicates that the domain structure is reflected in the growth morphology 26,27. On the basis of the X-ray diffraction and AFM results, we can construct the domain structure of Bi_2_Te_3_ films with respect to the GaAs substrate, as illustrated in Fig. 1d.

High-resolution, high-angle annular dark-field (HAADF) scanning tunnelling electron microscopy (STEM) analysis also confirms the epitaxial growth of (001) Bi_2_Te_3_ on the GaAs substrates, as shown in Fig. 1e. The low-magnification STEM image (left panel in Fig. 1e) shows dark strips indicating the van der Waals gap. The high-magnification STEM image (right panel in Fig. 1e) clearly shows the sequence of Bi (bright contrast) and Te (dark contrast) atoms in the quintuple layers (QLs)^28^. Figure 1f shows the periodic repetition of the intensity peaks corresponding to the white box marked in Fig. 1e (right panel). The Te (1)–Bi–Te (2)–Bi–Te (1) atoms and van der Waals gaps are clearly distinguished, where Te (1) has van der Waals bonding with the neighbouring Te (1) along the [001] direction, and Te (2) exhibits only covalent bonding.

**Figure 2:**
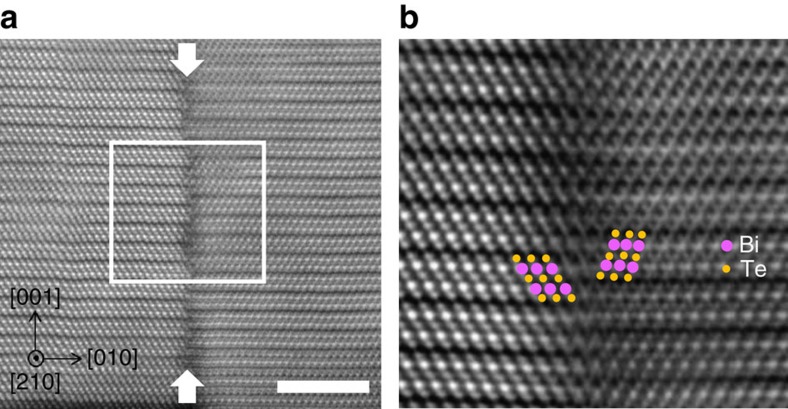
60° twin boundary of Bi_2_Te_3_ films. (**a**) High-resolution cross-sectional HAADF-STEM images of the 60° twin boundary along the [210] zone axis. The scale bar is 5 nm. The vertical twin boundary is indicated by two arrows. (**b**) Inversed Fourier-filtered images of the white box marked in (**a**). For clarity, the Bi atoms are depicted as purple circles and Te atoms are orange in colour. The atomic structure is mirrored relative to the vertical domain boundary.

The 60° twin boundary is revealed by the HAADF-STEM analysis in Fig. 2a. The interface of the domain boundary is clearly observed with marked arrows. Figure 2b shows the inversed Fourier-filtered image of the interface at the domain boundary. In this image, the 60° tilted domain boundary is clearly observed. The atomic structure is mirrored relative to the vertical domain boundary. For clarity, Bi atoms are depicted as purple circles, whereas Te atoms are orange in colour.

### Effect of 60° twin boundary on electrical transport property

To investigate the influence of the 60° twin boundary on the electrical properties, it is essential to control the boundary length. For the two-dimensional nucleation and growth mode, the domain types are determined by the nucleation stage. To increase the twin boundary length in a sample, each domain size needs to be decreased. It is well known that the domain size is inversely proportional to the driving force for nucleation: as the number of nucleation sites increase, the domain size decreases, hence a longer domain boundary. On the basis of this idea, we systematically control the growth rate of Bi_2_Te_3_ films from 0.5 to 1.2 nm min^−1^ and analyse the domain structure in real space using the electron backscattering diffraction (EBSD) technique.

**Figure 3:**
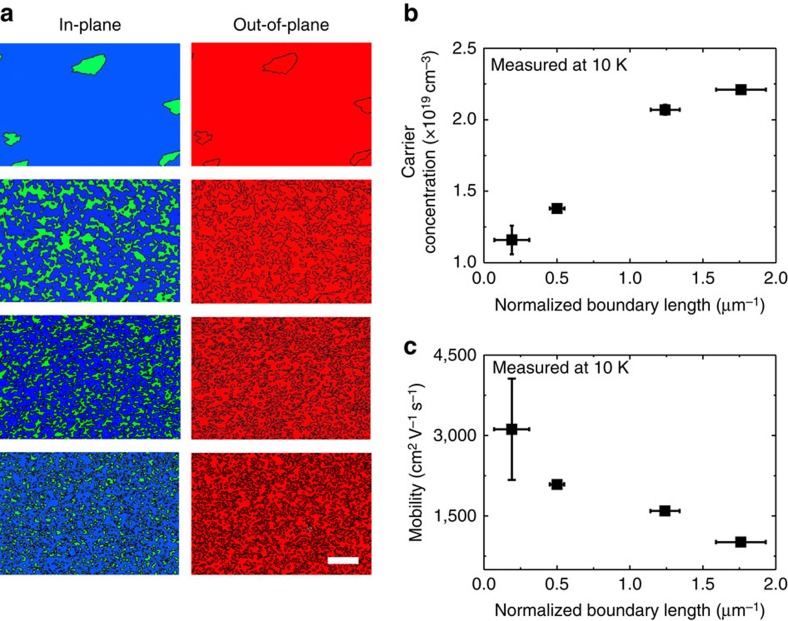
Domain controlled Bi_2_Te_3_ films and electrical properties. (**a**) In-plane (left) and out-of-plane (right panel) EBSD data of domain structure-controlled Bi_2_Te_3_ films. The blue and green areas in the in-plane EBSD images represent each domain with a 60° rotation along the *c*-axis. Their interface is the 60° twin boundary. Blue, green and red colours indicate the crystallographic orientations of [210], [120] and [001], respectively. The detailed colour orientation code is shown in Supplementary Fig. 3. Scale bar, 10 μm. (**b**) The carrier concentration and (**c**) mobility as a function of the normalized boundary length. The error bars are estimated by the measurements of three samples for each data point.

Figure 3a shows the in-plane (left column) and the out-of-plane (right column) EBSD images of the Bi_2_Te_3_ films grown at various growth rates. Note that the out-of-plane EBSD images show only a single red colour regardless of the growth rate, indicating that all of the samples have a (001) orientation along the *c*-direction. This is also consistent with the X-ray diffraction results in Fig. 1a. On the other hand, the in-plane EBSD images exhibit two distinct areas with green and blue colours, which stand for two different domains rotated by 60° to each other.

On the basis of the EBSD analyses, we can identify 60° twin boundaries by the borders between the green and blue areas in Fig. 3a. To estimate the twin boundary portion with respect to the total volume of the Bi_2_Te_3_ film, we normalize the boundary length by the total area in the EBSD images. Note that the boundary length determined by the EBSD analyses does not represent the absolute boundary length because it is dependent on the magnification of the EBSD image. Fine meanderings at an atomic level are not included as a boundary length at the low magnification of × 2,000 in Fig. 3a. However, as all of the images are measured at the same magnification, the normalized boundary length can be used for a relative comparison. The normalized 60° twin boundary length turns out to be proportional to the growth rate (Supplementary Fig. 2).

The electrical transport properties of these domain-engineered Bi_2_Te_3_ films are characterized by the Hall measurement using the van der Pauw method. To obtain the intrinsic carrier concentration, the electron concentration and mobility are measured at 10 K, as shown in Fig. 3b,c, respectively. The electron carrier concentration increases proportionally to the normalized length of the 60° twin boundary, while the electron mobility decreases. The latter is consistent with the previous concept that boundaries in a solid usually act as a defect to suppress the transport speed by scattering^10^. However, the former is an unexpected result. This indicates that the 60° twin boundary in Bi_2_Te_3_ can be a source of electron generation. As the density of the twin boundaries is controlled by the H_2_ flow rate, it is important to rule out other possible effects that can concomitantly occur during the H_2_ flow rate change. For example, potential candidates are the surface reduction effect^29^ and bulk doping by various defect formations^10^. We confirm that the free-electron carriers generated by these mechanisms are very small with a level of approximately 1 × 10^18^ cm^−3^ in our samples. Thus, we can conclude that the increase of carrier concentration is correlated with the density of the 60° twin boundaries. For details, see Supplementary Figs 3 and 4 and Supplementary Note 1.

### First-principle calculations

**Figure 4:**
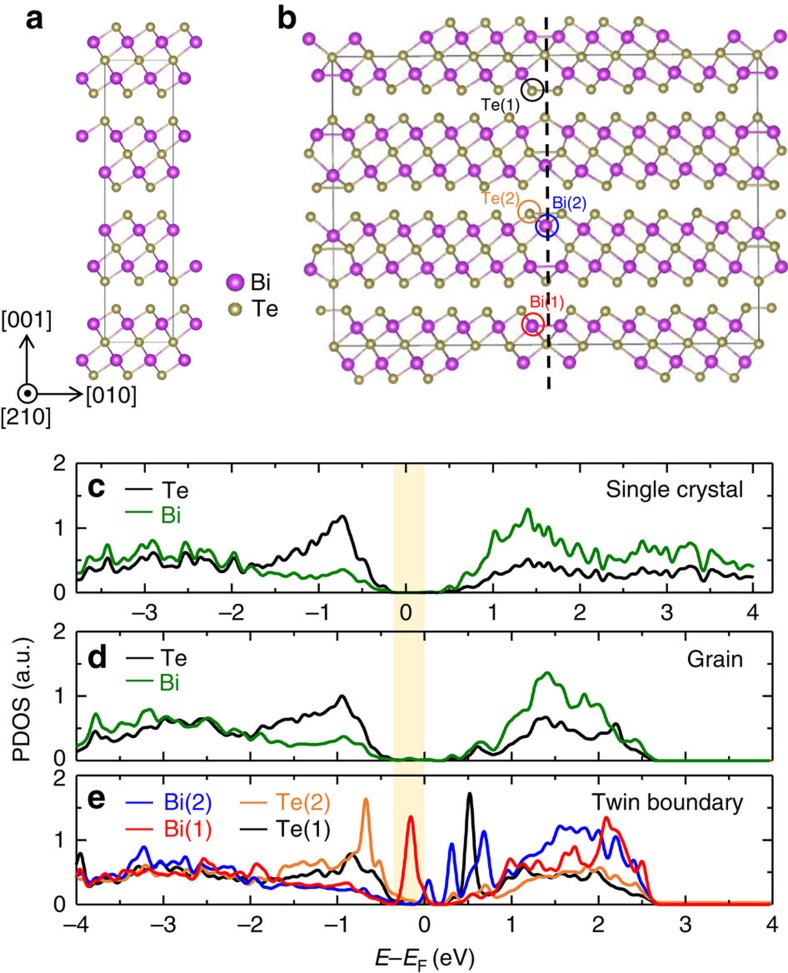
DFT computational simulation. Atomic structures of (**a**) the unit-cell of Bi_2_Te_3_ and (**b**) Bi_2_Te_3_ with a 60° twin boundary. The dotted black line indicates the 60° twin boundary. (**c**) The projected density of states (PDOS) of bulk Bi_2_Te_3_, (**d**) the grain region and (**e**) the boundary region (bottom panel). Four different atoms are chosen to plot the PDOS at the boundary region (as marked in **b**). Navy, orange, red and blue coloured circles correspond to Te (1), Te (2), Bi (1) and Bi (2) atoms, respectively. Three panels (**c**–**e**) are aligned to match the valence band maximum. The calculated band gap is indicated by the shaded area with the colour yellow.

To elucidate the physical origin of this experimental observation, we perform a first-principle calculation using the Vienna *ab-initio* simulation package^30^,^31^. Figure 4a shows the tetragonal unit-cell of Bi_2_Te_3_. To create a 60° twin boundary, one of the two 2 × 3 × 1 super-cells is rotated along the [001] axis by 60°, and then the two super-cells are attached to each other along the [010] axis with the same interval of the atomic layers, as shown in Fig. 4b. The super-cell with the twin boundary is relaxed with a free elongation along the [010] direction until the internal force becomes less than 0.02 eV Å^−1^. The reciprocal space is sampled by a grid of the Γ-centred 6 × 2 × 2 mesh in the Brillouin zone, where Γ is the (0, 0, 0) position in reciprocal space. In the bulk region, the nearest bond lengths of Bi–Bi and Te–Te are 4.41 and 4.44 Å, respectively. On the other hand, in the twin boundary region, the interatomic distances change to accommodate the structural misfit. Mostly, the bonding distances shorten, ranging from 3.11 to 3.54 Å.

The projected density of states (PDOS) is plotted in Fig. 4c–e, where the three panels are presented in a way to align the valence band maximum to identify the band gap. The band-gap of bulk Bi_2_Te_3_ (as Fig. 4a) in the top panel is calculated to be 0.36 eV. This value is nearly the same as previous theoretical results^32^, although larger than the experimental band-gap (approximately 0.15 eV)^10^. This is because spin-orbit-coupling is not applied for the efficiency of the calculations. As verification, we confirm that the shape of the PDOS of the bulk Bi_2_Te_3_ with spin-orbit-coupling (not shown) is nearly the same as that without spin-orbit-coupling, as also reported elsewhere^33^. The PDOS of the bulk-like region of Bi_2_Te_3_ in the structure having the 60° twin boundary (as Fig. 4b) in Fig. 4d is similar to that in Fig. 4c, implying that the electronic properties of this region are nearly similar to those of the bulk.

On the contrary, the PDOS for the 60° twin boundary region in Fig. 4e is significantly different from that of bulk in that several states are generated in the intrinsic band-gap. Four different atoms (as marked in Fig. 4b) are chosen to plot the PDOS at this boundary. It is noted that newly occupied states are formed by the Bi(1) atom owing to the shortening of the Bi–Bi distance (3.11 Å). When the shortened bond of Te(2)–Te(2) (3.54 Å) forms, newly occupied levels inside the band-gap are also generated. In addition, the Bi(2) atom, which is the first nearest neighbour of the Te–Te bond, generates newly unoccupied levels near the conduction band minimum (CBM).

## Discussion

Our control of the population of the 60° twin boundaries in (001) epitaxial Bi_2_Te_3_ thin films and the characterization of the structural and electrical properties allow us to evaluate the twin boundary's effects on the electrical transport properties. Our results show that the 60° twin boundary can create free electron carriers and suppress the transport mobility. Given that the twin boundary is usually regarded as one of the least disordered grain boundaries, it is surprising that it can significantly reduce the electron mobility. This might provide insight into the reason why the conventional nano-structuring approach successfully demonstrated in *p*-type thermoelectric materials does not work in *n*-type ones. It appears that the electron mobility is very sensitive to a small distortion of atomic bonds.

The fact that free electrons can be created at the 60° twin boundary is also remarkable. This arises from the electronic structure modification at the boundary because of the changes in the atomic bonds to accommodate the structural misfit. On the basis of DFT calculations, two effects can possibly cause the creation of free electrons at the 60° twin boundary. First, the newly formed occupied states within the intrinsic bandgap may play the role of donor-doping levels; although it is difficult to precisely determine whether they are donors or acceptors because they are close to both the valence band maximum and the CBM, spreading the whole range of the band-gap. Second, the enlarged DOS near the CBM can also be responsible for the increase in the electron carrier concentration. Therefore, we can obtain a physical insight into the mechanism of the free-electron generation at the 60° twin boundary: the distortion of atomic bonds at the boundary can cause a defect level, potentially working as a donor, as well as the enlarged DOS near the CBM (Supplementary Fig. 5 and Supplementary Note 2).

Our work suggests the concept of structural doping, where electrical doping can be achieved by a structural modification rather than an impurity doping. It may be possible to enhance the transport property by preserving the high quality channel region from the defective doping region through the alignment of twin boundaries, which is similar to the origin of the high mobility in a two-dimensional electron gas. Our experimental approach and results offer insight into the electrical properties in other layered chalcogenide materials, such as MoS_2_, where it is crucial to control the electrical transport behaviour as a channel layer of next-generation transistors. Moreover, our work provides excellent opportunities to explore undiscovered physical properties using an interface-engineering approach on common materials.

## Methods

### Bi_2_Te_3_ film growth

Bi_2_Te_3_ films were grown on GaAs substrates using metal-organic chemical vapour deposition technique with a horizontal flow reactor at atmospheric pressure. In this work, a (111) GaAs wafer is used as the substrate for the growth of Bi_2_Te_3_ films. The three-fold symmetry of the (111) GaAs surface is well-matched with that of the (001) Bi_2_Te_3_ surface. Before loading the substrate into the reactor, we cleaned the GaAs substrates with HCl (18%) for 3 min and rinsed them in deionized water. The growth temperature was fixed at 360 °C (ref. 22). The Pd-filtered high purity H_2_ was used as the reactant and carrier gas. Tri-methylbismuth (Bi(CH_3_)_3_, TMBi) and di-isopropyltellurium (Te(C_3_H_7_)_2_, DIPTe) were used as the sources of Bi and Te, respectively. To maintain the constant partial pressure and avoid premature decomposition, the TMBi and DIPTe were kept in constant temperature baths at 0 and 25 °C, respectively. The growth rates were controlled from 0.5 nm min^−1^ to 1.2 nm min^−1^ by the variation of the flow rate of the H_2_ carrier gas for TMBi (10–30 s.c.c.m.) and DIPTe (245–735 s.c.c.m.), respectively. The precursor ratio of DIPTe:TMBi was fixed at 9 for stoichiometric Bi_2_Te_3_ films. The growth time varied from 2,500 to 6,000 s to obtain equivalent film thickness.

### Characterizations

A high-resolution X-ray diffractometer (HRXRD, X'PertPro, PANalytical, the Netherlands) equipped with a (220) Ge crystal 4-bounce hybrid monochromator (*λ* (wave length)=1.5406 Å, 30 kV, 10 mA) was used. High-resolution transmission electron microscopy was carried out using FEI's Titan 80-300 microscope operating at 300 keV for the analysis of the crystal structure. Domain structure analyses were also performed using electron backscatter diffraction (EBSD, Hitachi S-4300) with Bruker e-Flash software. The measurements of electrical properties were performed in a commercial cryostat (CTI-Cryogenics, 8200 compressor) with a Keithley 220 current source and a Keithley 182 nano-voltmeter. The conventional van der Pauw method was used to evaluate the carrier concentration and mobility of the films.

### Computational methods

All of the calculations were performed using the Vienna *ab-initio* simulation package^30^,^31^. The projector-augmented wave method^34^ within the generalized gradient approximation^35^ was used with cut-off energy of 500 eV. Valence electron configurations with *s*^2^*p*^3^ and *s*^2^*p*^4^ were used for the pseudo-potentials of Bi and Te atoms, respectively. For unit-cell optimization, the tetragonal cell of Bi_2_Te_3_ with a space group of R
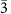
m was prepared by transformation from the conventional hexagonal unit-cell. The unit-cell was fully relaxed until the internal force became less than 0.005 eV Å^−1^. The projected density-of-states were obtained by using Gaussian smearing with a width of 0.05 eV. A 6 × 6 × 6 Γ-centred *k*-grid mesh was used. The VESTA program was used to draw the atomic structures and to calculate the interatomic distances^36^.

### Data availability

The data that support the findings of this study are available from the corresponding authors on request.

## Additional information

**How to cite this article:** Kim, K.-C. *et al.* Free-electron creation at the 60° twin boundary in Bi_2_Te_3_. *Nat. Commun.* 7:12449 doi: 10.1038/ncomms12449 (2016).

## Supplementary Material

Supplementary Information Supplementary Figures 1-5 and Supplementary Notes 1-2
