# Supplementary material for: Free-electron creation at the 60° twin boundary in Bi2Te3
Source: Nat Commun. 2016 Aug 16;7:12449. doi: 10.1038/ncomms12449 (PMC4990697; doi:10.1038/ncomms12449)
Supplement: Supplementary Information — Supplementary Figures 1-5 and Supplementary Notes 1-2 [file ncomms12449-s1.pdf]

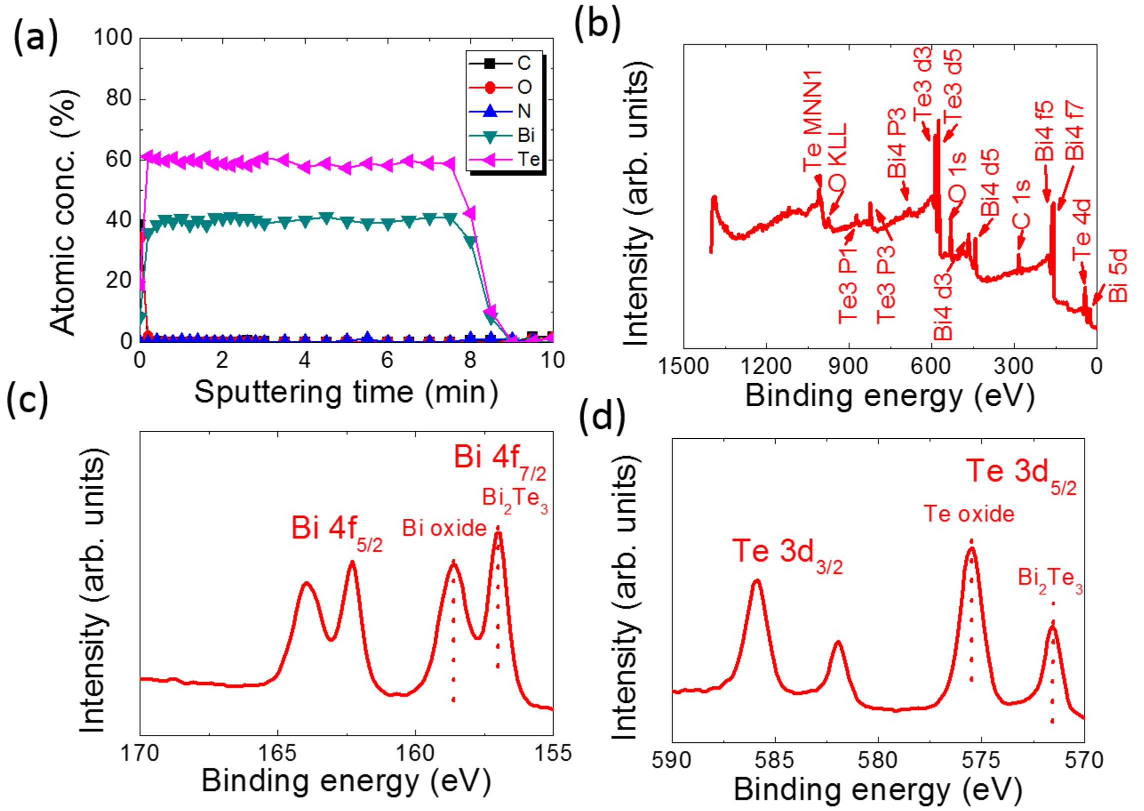

Supplementary Figure 1. (a) Auger electron spectroscopy (AES) depth profiles of Bi_2Te_3 films. A composition ratio of Bi:Te in the AES depth profiles is very close to 2:3, indicating that all the films are stoichiometric. (b)-(d) shows the X-ray photoelectron spectroscopy analysis of wide scan, Bi 4f, Te 3d core levels, respectively. The peaks from the oxides are ascribed to the thin oxide surface layer, which was formed by the air exposure of the samples as shown in (c)-(d).

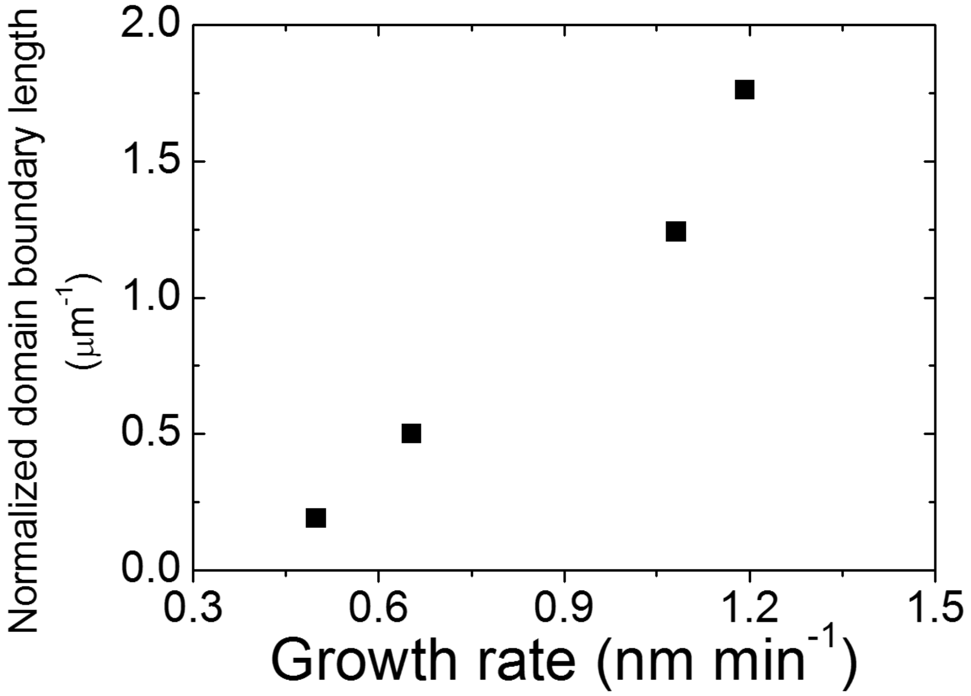

Supplementary Figure 2. Control of domain boundary length by the growth rate of Bi_2Te_3 films.

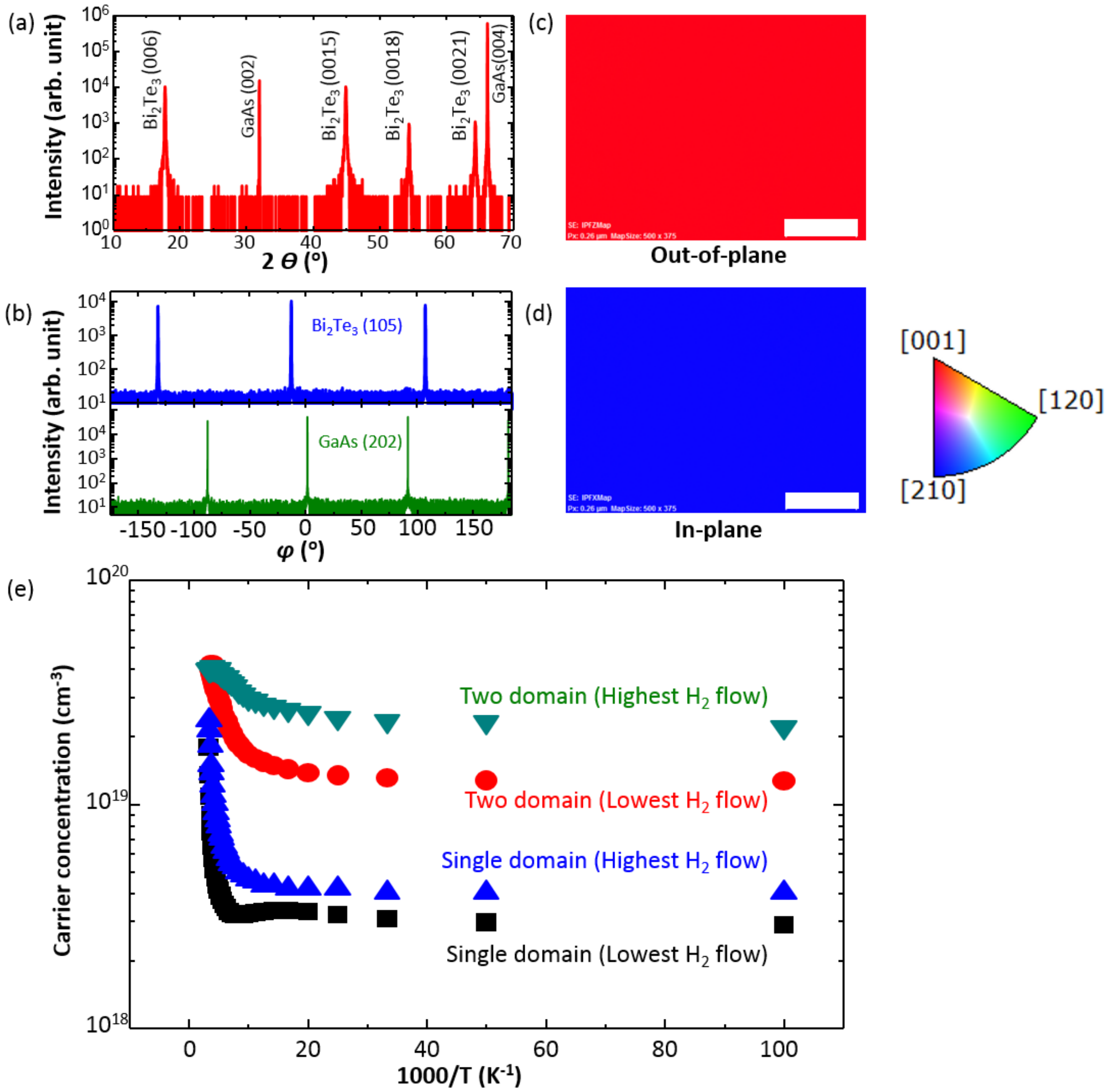

Supplementary Figure 3. (a) XRD out-of-plane θ-2θ scan, (b) in-plane φ scan, (c) out-of-plane EBSD, and (d) in-plane EBSD images of the (001) single-domain Bi_2Te_3 thin film on (001) GaAs substrate with 4° miscut. (e) Low-temperature measurement of the carrier concentration of various Bi_2Te_3 thin films grown different H_2 flow and different domain structures. Bi_2Te_3 films grown on (001)-oriented, 4°miscut GaAs substrate are all single-domain, regardless of the H_2 flow rate. On the other hand, Bi_2Te_3 films grown on (111) GaAs substrate have two domains with 60° rotation, and the density of the twin boundary increases with H_2 flow rate, as shown in the manuscript. Scale bar is 30 μm

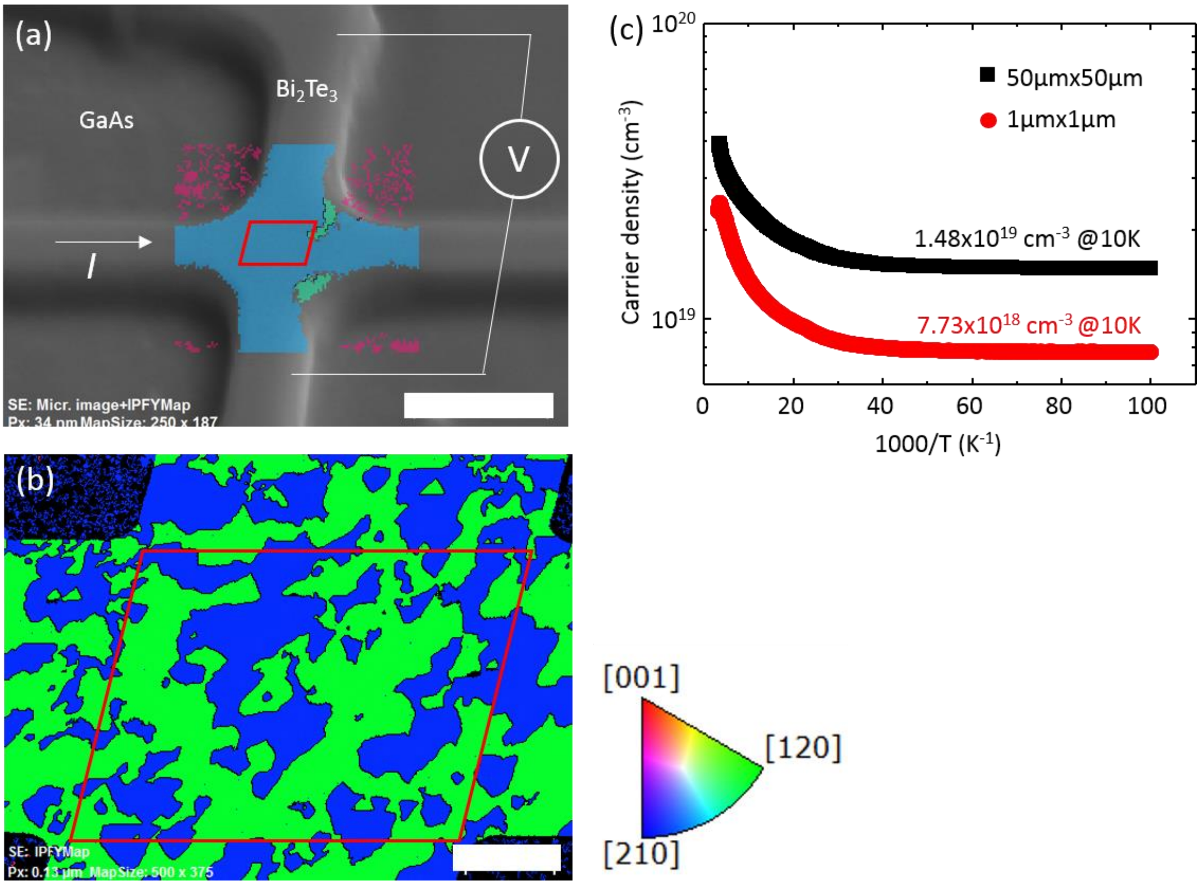

Supplementary Figure 4. Hall bar patterns of (a) 1 μm × 1 μm and (b) 50 μm × 50 μm size. The red area is the detection region. The EBSD image is integrated in SEM image in Fig (a). (c) Low-temperature measurement of the carrier concentration in two different-sized Hall bar of the same sample. Scale bars in (a) and (b) are 2 μm and 10 μm, respectively.

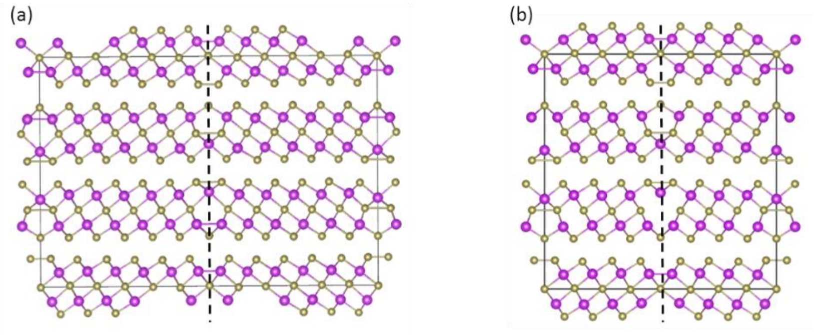

Supplementary Figure 5. . Two different sizes of supercells for carrier density comparison.

Supplementary Note 1.

It is necessary to rule out other possible scenarios affecting the increasing carrier concentration of our Bi_2Te_3 thin films with the increasing H_2 flow rate (growth rate). , the other possible candidates are the reduction effect of the film surface and the bulk doping effect through the formation of point defects by the different growth rate.

We measured the carrier density of an isolated single-domain area in two ways: by fabricating single-domain Bi_2Te_3 films grown under the same MOCVD growth condition, and by fabricating a micro-Hall bar pattern to access a single-domain region in the original two-domain sample. In the below, experimental results on these two cases will be discussed.

Twin-free, single-domain Bi_2Te_3 films

We were able to fabricate twin-free, single-domain, epitaxial (001) Bi_2Te_3 thin films using 4° miscut (001) GaAs substrate. The other growth conditions of MOCVD are all the same as we used to grow samples shown in the main text. Due to the unique van der Walls epitaxy nature of Bi_2Te_3 material, (001) epitaxial Bi_2Te_3 films with 3-fold symmetry can be grown on (001) GaAs substrate with 4-fold symmetry, which is not possible in the normal epitaxial films. Moreover, the vicinal surface of 4° miscut (001) GaAs substrate allows us to grow a single-domain, twin boundary-free, epitaxial Bi_2Te_3 film. Supplementary Figure 3a and b show the out-of-plane θ-2θ scan and the azimuthal φ scan of (105) plane of Bi_2Te_3 film as well as (202) plane of GaAs substrate, respectively. These results indicates that the (001) epitaxial Bi_2Te_3 film is grown with a single domain structure, i.e. without twin boundaries. The out-of-plane and in-plane EBSD images also confirm that the Bi_2Te_3 film grown on 4° miscut (001) GaAs substrate is twin-free as shown in supplementary Figure 3c and d, respectively. Such a single-domain structure is maintained for all H_2 flow rates we used to control the growth rate.

Supplementary Figure 3e shows the temperature-dependent carrier concentration of the twin-free (001) Bi_2Te_3 film grown at the two extreme H_2 flow rates. It is noted that the variation of the carrier concentration of them is very small with a level of approximately1×10^{18} cm^{-3}. This indicates that other potential mechanisms such as the surface reduction effect and the bulk doping by the point defect generation by the H_2 flow variation cannot explain our observation of the carrier density changes with a level of approximately 1×10^{19} cm^{-3}.

Micro-Hall bar pattern measurement on a single-domain region in the two-domain sample

We also compared the carrier density of the following cases in the same sample: a large area including twin boundaries and a small region without boundaries, *i.e.*, a single-domain area. For this comparison, we patterned the two different-sized Hall bars with 1 μm × 1 μm and 50 μm × 50 μm on the same sample as shown in Supplementary Figure 4a and b, respectively. EBSD image of the 1 μm × 1 μm Hall bar (Supplementary Figure 4a) shows that there exist no 60° twin boundary within the square area. On the other hand, the 50 μm × 50 μm Hall bar contains the 60° twin boundaries (Supplementary Figure 4b). The low-temperature measurement shows that the electron carrier density at 10 K of the single-domain region is 7.73 $\times 10^{18}$ cm^{-3} while that of the area having twin boundaries is 1.48$\times 10^{19}$ cm^{-3}. This is a direct evidence that the 60° twin boundary in Bi_2Te_3 can be a source of free electrons.

In conclusion, we directly showed that the carrier concentration of the twin-free, single-domain Bi_2Te_3 is much lower than that of the Bi_2Te_3 having twin boundaries. We have shown this by two different ways: using the as-grown, single-domain sample, and using a selectively-patterned, single-domain region in the two-domain sample. Our results clearly exhibit that other possibilities such as the surface reduction effect and the bulk doping effect via non-stoichiometry and point/antisite defects do not affect our conclusion that the free electron carriers can be created at the 60° twin boundary in Bi_2Te_3, as their contribution is low.

Supplementary Note 2. Carrier concentration calculation based on DFT results

Carrier concentration of n-type semiconductor can be calculated as the equation (1)

$$n = \int_{N_{CBM}}^{\infty} N_c\{1 + \exp(\frac{E - E_F}{k_B T})\}^{-1} \quad (1)$$

, where N_C is the density of state near the conduction band minimum (CBM), and $\{1 + \exp\left(\frac{E-E_F}{k_B T}\right)\}^{-1}$ is the Fermi function. The carrier concentration is proportional to the size of DOS at the CBM. To obtain the carrier concentration from the DFT results, the following aspects have to be first considered: our Bi_2Te_3 samples are naturally doped in the level of approximately 3×10^{18} cm^{-3} at 10 K as shown in supplementary Figure 3e. This belongs to the extrinsic region where the carriers are determined by the extrinsic doping. Thus, this level of carriers should be the base carrier concentration of the single-domain Bi_2Te_3. To obtain such level of carrier concentration in single-domain Bi_2Te_3, we shifted the E_F over the CBM to the same position derived in the single-domain supercell. As the exact E_F cannot be determined by the DFT calculation, this assumption is inevitable, however, this makes it difficult to directly compare the calculated carrier density with the experimental one. Therefore, we investigated the trend of carrier density change with the twin boundary density in the calculations.

We carried out the same DFT calculations on two-different-sized supercells, as shown in supplementary Figure 5. The supercell ***b*** has a shortened length by 2/3 along x-direction compared to the supercell ***a***. Thus, the density of twin boundary in the supercell ***a*** (632.1 μm^{-1}) is 33% larger than that in ***b*** (424.9μm^{-1}). The calculated carrier concentration of the supercell ***a*** is approximately 1.30×10^{20} cm^{-3} while that of the supercell ***b*** is approximately 1.03×10^{20} cm^{-3}. This indicates that the carrier concentration of Bi_2Te_3 is proportional to the density of the twin boundary, which is the same trend as observed in the experiment.
